# Importance of the type of pharmacological treatment in patients with severe mental disorder

**DOI:** 10.1192/j.eurpsy.2024.1453

**Published:** 2024-08-27

**Authors:** M. Lucas, P. Romero, N. Sirvent, R. Roig, J. Bajén, M. Aliño, C. Escobar, C. López

**Affiliations:** PSYCHIATRIC, SESCAM, ALBACETE, Spain

## Abstract

**Introduction:**

The use of long-acting treatments is a common clinical practice in psychiatry. No disease insight and the risk of treatment discontinuation in a significant portion of our patients, increase the demand for psychiatric emergency and hospital admissions. Treatment adherence must be facilitated, taking into account possible side effects and patient´s subjective satisfaction.

**Objectives:**

-Evaluate the type of long-acting intramuscular treatment in selected patients. -Evaluate the differences in treatment satisfaction between different types of long-acting intramuscular treatments as well as frequency of psychiatric emergency and hospital admissions in the last year.

**Methods:**

We select patients with different severe mental disorders who stay in a Medium Stay Unit, Sociosanitary Community Residence, Supervise house and Residence for the elderly in Albacete (Spain); all of them, with intramuscular neuroleptic treatment (zuclopenthixol dihydrochloride, aripiprazole long acting, palmitate paliperidone monthly, 3-monthly and 6-monthly) at least 1 year.

We evaluate their sociodemographic characteristics, the satisfaction questionnaire with the treatment (TSQM-9) and the rate of psychiatric emergencies and admissions after current intramuscular treatment in last year.

**Results:**

We have selected 57 patients with an average age of 45.86. 78.94% with a diagnosis of schizophrenia, 12.28% with schizoaffective disorder, 5.26% bipolar disorder and 3.5% unspecified psychotic disorder.

We can see in the graphics below that the longer duration of the intramuscular treatment, the greater satisfaction in all the items of the TSQM-9 questionnaire.

31% of the patients with zuclopenthixol dihydrochloride treatment, have gone to psychiatric emergencies and 28% of psychiatric admissions in the last year.18% of the patients with aripiprazole long acting, 17% with paliperidone palmitate long acting-monthly and 12% de 3-monthly have gone to psychiatric emergencies and 15%, 12% and 12% needed psychiatric admissions respectively. Patients with palmitate long acting-monthly have not emergencies or psychiatric admissions in the last year.

**Image:**

**Figure:**
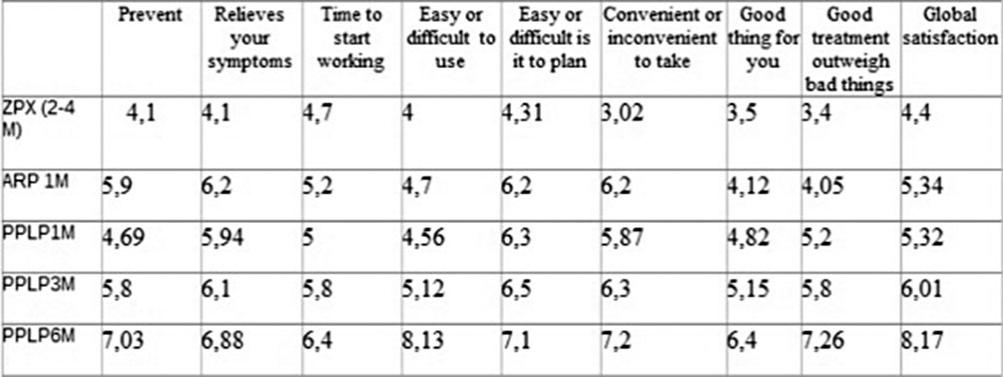

**Image 2:**

**Figure:**
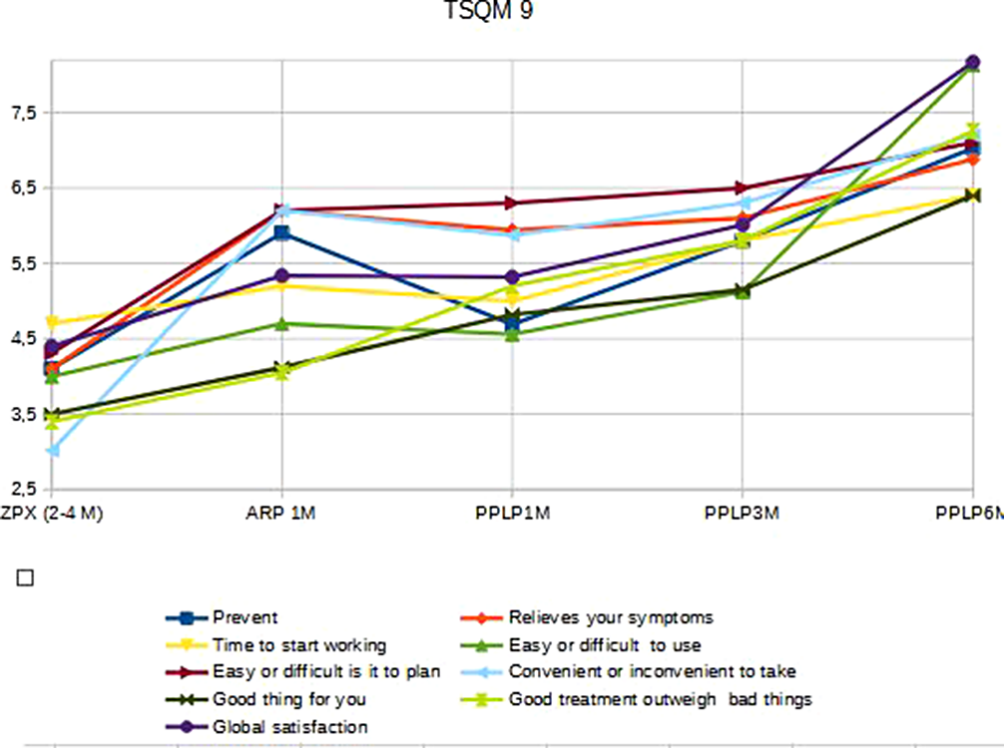

**Image 3:**

**Figure:**
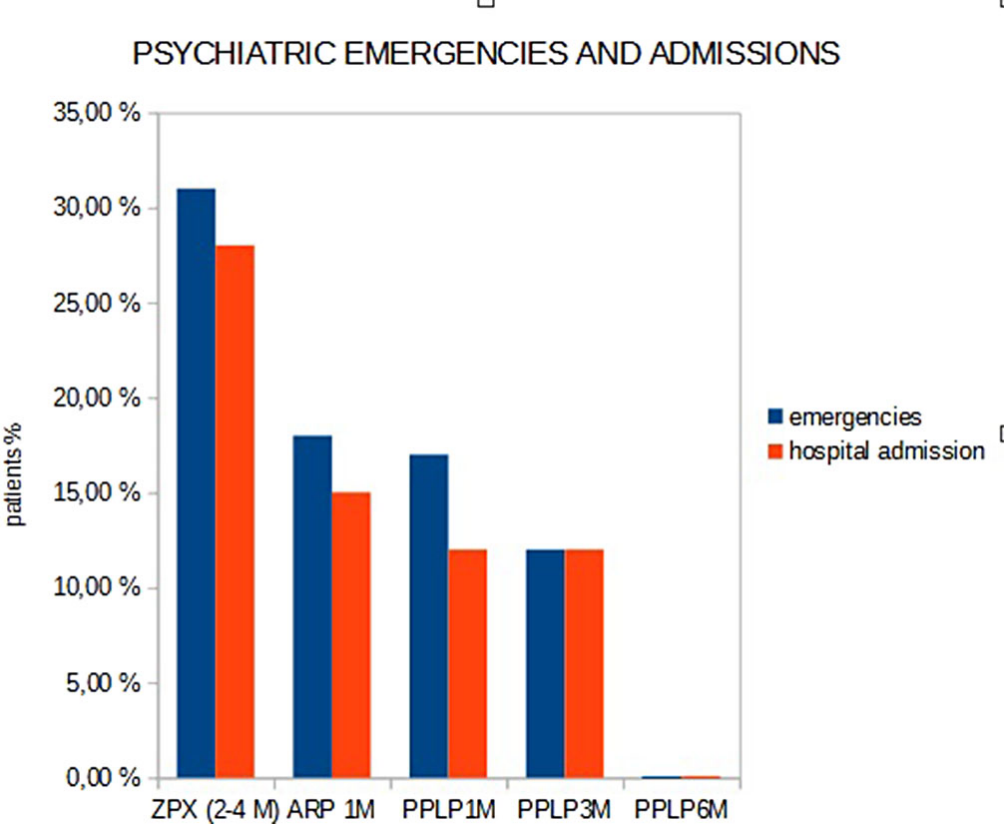

**Conclusions:**

- The longer long acting of the intramuscular treatments, the better patient satisfaction.
- With the longer duration treatment (Palmitate paliperidone LD 6 month), we have lower psychiatric emergencies and hospital admissions.

**Disclosure of Interest:**

None Declared

